# Supplementary material for: Trametinib-Induced Epidermal Thinning Accelerates a Mouse Model of Junctional Epidermolysis Bullosa
Source: Biomolecules. 2023 Apr 25;13(5):740. doi: 10.3390/biom13050740 (PMC10216840; doi:10.3390/biom13050740)
Supplement: Supplementary file 1 [file biomolecules-13-00740-s001.zip › biomolecules-2346837-supplementary.pdf]

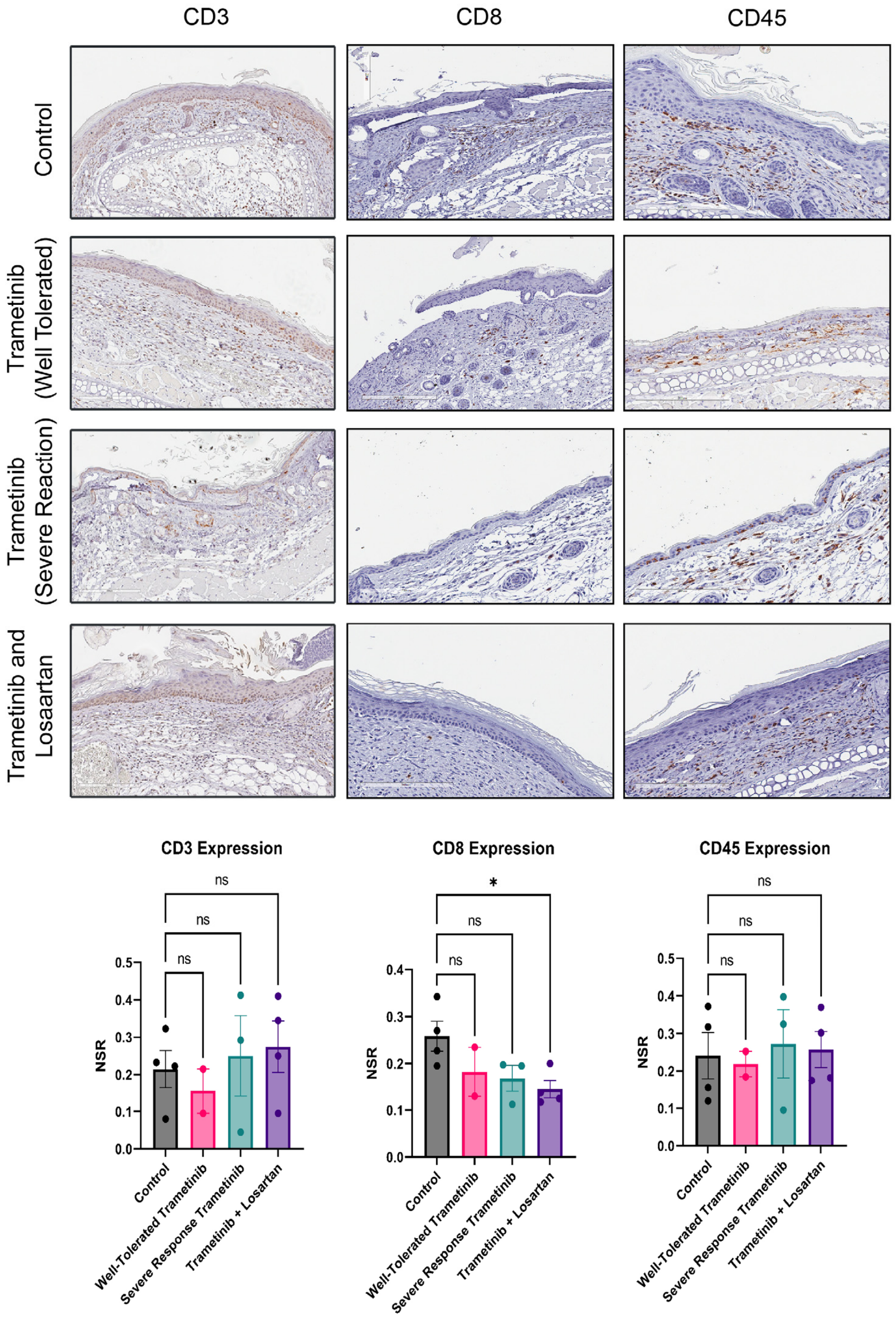

Supplementary Figure S1. Trametinib and Losartan treatment targets CD8. Representative images (top) of IHC staining for CD3 (left), CD8 (middle), and CD45 (right). Graphs showing mean±SEM (bottom) of NSR values for CD3, CD8, and CD45 expression with Kruskal-Wallis test performed (Dunn's correction) for CD3 and CD45 and one-way ANOVA analysis performed for CD8 (Dunnett's correction). Trametinib and Losartan co-treatment significantly inhibits CD8 expression (*: $p \leq 0.05$).
